# Cognitive Robustness in Dermatopathology—Diagnostic Thinking Beyond Rules and Routines

**DOI:** 10.1111/cup.14861

**Published:** 2025-09-04

**Authors:** Cornelia Sigrid Lissi Müller

**Affiliations:** ^1^ MVZ for Histology, Cytology and Molecular Diagnostics Trier Trier Germany; ^2^ Faculty of Medicine Saarland University Homburg/Saar Germany

**Keywords:** cognitive robustness, dermatopathology, diagnostic reasoning, error culture, uncertainty management

Dermatopathological diagnosis relies on structured pattern analysis, ensuring reproducibility, communication, and quality. Yet in practice, many lesions resist clear classification due to heterogeneity, artifacts, or overlapping features. Strict adherence to rules can then obscure diagnostic uncertainty. In complex cases, experienced dermatopathologists often rely on intuitive, experience‐based recognition—grasping patterns at a glance and identifying relevant deviations early. This aligns with the Recognition‐Primed Decision model, where familiar patterns trigger plausible diagnostic pathways without exhaustive comparison, enabling efficient and well‐grounded judgments even under pressure [1].

Current work on clinical thinking emphasizes that diagnostic expertise is not based exclusively on analytical, conscious processing or purely intuitive processes. Rather, both thinking strategies—analytical and non‐analytical—should be used flexibly and context‐dependently, depending on the complexity of the findings and diagnostic uncertainty [2]. In dermatopathological practice, this means that neither intuition alone nor exclusively rule‐based processing guarantees a valid diagnosis. The decisive factor is the ability to switch between the two modes of thinking as appropriate to the situation and to combine them in a targeted manner. Despite the recognized importance of intuitive decision‐making processes for medical diagnosis, their specific relevance for dermatopathological diagnosis has so far been largely ignored [2, 3].

While the role of intuitive pattern recognition is increasingly being researched and discussed in radiology or intensive care medicine [4, 5, 6], a rationalized, rule‐based discourse continues to dominate in dermatopathology. The importance of subjective, cognitively influenced decision‐making processes usually remains implicit—although they are central to diagnostic action.

The concept of *cognitive robustness* describes the ability to make consistent and reliable decisions even under conditions of uncertainty, incompleteness, or ambiguity. It originally comes from decision psychology and cognitive research and has recently been taken up in medical diagnostics—particularly in radiology, emergency medicine, and anesthesia—as an explanatory concept for the performance of experienced diagnosticians under complex conditions [7, 8, 9].

Unlike purely intuitive decisions, cognitive robustness relies on a stable internal structure of diagnostic knowledge built through experience, pattern formation, and feedback. It allows valid decisions even when classifications fail, findings conflict, or data is missing—drawing on implicitly available, cognitively consolidated expertise that often outpaces conscious analysis [2, 9].

Cognitive robustness can be seen, for example, in the ability to recognize subtle, non‐obvious changes as pathological, to integrate multiple biopsies of a patient in an internal comparison, or to intuitively recognize a disharmonious detail in an overall histological picture as diagnostically relevant. It is about a kind of *robust flexibility* in diagnostic thinking: the ability to quickly recognize known patterns—but also the openness to consciously question and recalibrate one's own judgment in the event of deviations [3, 10]. This competence is particularly important when morphological criteria are borderline or ambiguous, technical artifacts complicate the findings, clinical‐anamnestic additional information is missing or contradictory, or several possible differential diagnoses come into question. In dermatopathological practice, this applies to melanocytic lesions, early cutaneous lymphomas, regressive tumors, or inflammatory dermatoses—that is, findings with a high degree of complexity and variable clinical and histopathological presentation [11, 12, 13].

Unlike everyday intuition, cognitive robustness is a learnable skill. It develops through repeated exposure to challenging cases, reflective practice, feedback, and active engagement with uncertainty—going beyond rule application and enabling systematic professional growth [14, 15].

## 1 Cognitive Robustness at the Microscope—Observations From Dermatopathological Practice

In dermatopathology, many decisions emerge within seconds—an initial glance often triggers a diagnostic impression. This “pre‐sense” reflects a deeply ingrained, experience‐based visual comparison process and exemplifies cognitive robustness: forming a plausible hypothesis despite ambiguous findings. Such robustness depends less on age than on reflective practice, feedback, and tolerance of uncertainty. While experienced diagnosticians draw on broader pattern libraries, they are not immune to routine bias. With training, even younger colleagues can show high metacognitive flexibility. This approach is less rule‐bound than dynamic—balancing early pattern recognition with deeper analysis when expected features do not align [2, 5, 9, 10].

Cognitive robustness is demonstrated in such situations by the following characteristics:

– *Tolerance of ambiguity*: Experienced diagnosticians initially tolerate diagnostic uncertainty without making hasty categorizations. They recognize the relevance of ambiguity as a diagnostic signal [2, 4].
– *Internal referencing*: Instead of relying exclusively on external morphological criteria, they use their “internal archive” of comparable cases to classify patterns. This applies in particular to multiple biopsies from the same patient or rare constellations [10].
– *Dynamic hypothesis formation*: Diagnostic judgments are not made in a linear fashion, but as changing hypotheses that are continuously “negotiated” with the specimen. Changing the magnification, additional staining, immunohistopathology, or revisiting a previously observed area play a central role in this process [2, 9].
– *Contextualization of the image*: The histological observation is always related to the clinical context, the age of the patient, the localization, or known previous findings—even if this information is not immediately visually present. They are “thought through” [4, 9].
– *Resilience to formal incongruence*: Experienced dermatopathologists remain diagnostically stable, even if individual criteria are contradictory (“conflicting criteria”). They accept morphological ambiguities as part of biological reality—and make decisions not despite but based on this ambiguity, as Roncati et al. exemplify in their classification of unclear melanocytic lesions as SAMPUS, MELTUMP, or THIMUMP [10, 15, 16].

This form of diagnostic stability is not only an expression of individual experience, but also a result of metacognitive maturity: knowledge of one's own cognitive processes, the conscious regulation of uncertainty and trust in one's own implicit judgment. It distinguishes beginner diagnostics—which are strongly rule‐oriented and often dualistic—from the expert level, where uncertainty is part of the diagnostic repertoire [2].

This dimension has hardly been addressed in dermatopathology teaching to date. Training formats predominantly focus on learning defined criteria, the application of algorithms, or the supposed certainty provided by additional immunohistochemical or molecular diagnostics. The development of cognitive robustness, on the other hand—as an independent skill—is largely left to the individual learning path.

## 2 Cognition‐Based Decision Making—System 1 and System 2 in Dermatopathology

The distinction between two types of thinking—a fast, intuitive mode (“System 1”) and a slow, analytical mode (“System 2”)—goes back to the psychologist Daniel Kahneman and has significantly influenced cognitive research. In medical diagnostics, this model describes two complementary processes: the rapid recognition of familiar patterns and conscious, rule‐based deliberation in new or complex situations [17]. System 1 operates quickly, intuitively, and without conscious effort—as when experienced dermatopathologists instantly recognize a pattern before explicitly naming its features. In contrast, System 2 is slow, analytical, and rule‐based, guiding decisions in ambiguous or novel cases through detailed examination, classifications, and additional studies. These systems are complementary: System 1 generates initial hypotheses, whereas System 2 evaluates them. Expert diagnosticians shift fluidly between both modes. Cognitive robustness means not relying solely on intuition, but knowing when to engage each system—and when to switch [4, 9, 17]. Metacognitive control is key to dermatopathological expertise. Diagnostic errors often stem not from lack of knowledge, but from an imbalance between intuition and analysis—too much intuition risks premature closure; too much analysis may cause delays or overdiagnosis. Bernard Ackerman stressed the need to integrate both modes of thinking. He promoted a pragmatic, clinically relevant approach centered on “low‐power diagnosis”—recognizing patterns at low magnification through experience and intuition. His philosophy underscores the value of combining knowledge, critical thinking, and reflective judgment to manage uncertainty and reduce errors [18, 19].

Kahneman's model—with its distinction between intuitive (System 1) and analytical thinking (System 2)—offers a formative introduction to the understanding of diagnostic cognition [17]. However, there are other theories that represent valuable additions or alternatives, particularly for dermatopathology. These models are presented in Table 1. They help to understand diagnostic thinking in a more differentiated way and to describe the special features of microscopic decision making more precisely.

**TABLE 1:** Comparison of selected theoretical models of cognitive decision making in dermatopathology.

Model	Core concept	Relevance to dermatopathology	Relevance to cognitive robustness
Dual process theory (Kahneman) [17]	Two systems: fast, intuitive thinking (System 1) vs. slow, analytical thinking (System 2)	Intuitive pattern recognition vs. rule‐based analysis under the microscope	Cognitive robustness = flexible, context‐dependent switching between both systems
Dreyfus model of skill acquisition [15]	Experts progress from rule‐based behavior to experience‐based decision making	Novices rely on defined criteria, while experts intuitively perceive disharmonious patterns	Robustness develops in the upper stages: from “competent” to “expert”
Recognition‐primed decision (Klein) [1]	Decisions are based on rapid recognition of familiar patterns—without explicit comparison	“This looks like a…”: quick matching of current case with stored experience	Robust decisions through implicit pattern matching and evaluation of a few key features
Illness scripts/script theory [20, 21]	Mental “scripts” with typical disease constellations aid in case comparison	Comparison with stored visual‐clinical images (e.g., classic basal cell carcinoma)	Cognitive robustness = flexible application and adaptation of such scripts
Cognitive continuum theory (Hammond) [22]	Decision making exists on a continuum between intuition and analysis	Range from “clear melanoma at first glance” to “diffuse lichenoid interface pattern with broad differential”	Robustness = situationally appropriate placement along this continuum
Naturalistic Decision Making (NDM) [23]	Real‐world decisions under uncertainty, time pressure, and incomplete information	Everyday scenarios in pathology (e.g., no clinical data, time constraints, diagnostic ambiguity)	Robust decision making even under suboptimal conditions

*Note:* The following table provides a structured overview of key theoretical frameworks that describe cognitive processes in medical decision making, particularly as applied in radiology, pathology, and emergency medicine. These models offer complementary perspectives on diagnostic thinking—from rapid, experience‐based pattern recognition to gradual competence development and real‐world decision making under uncertainty. Each model is summarized with regard to its core concept, its specific relevance to dermatopathology, and its contribution to the concept of cognitive robustness. Together, they demonstrate that cognitive robustness is not bound to a single framework. Rather, it emerges as a higher‐order competence that integrates intuitive insight, analytical reasoning, and metacognitive control in a flexible, situation‐sensitive manner. In dermatopathology—an image‐based discipline often confronted with ambiguous patterns and incomplete clinical information—this capacity to shift between intuitive and structured reasoning is especially critical. The theories listed here provide a conceptual foundation for understanding, discussing, and—at least in part—teaching such diagnostic expertise.

These theories illustrate the multidimensional nature of diagnostic thinking—balancing intuition and analysis, rules, and experience. Dermatopathology, as a visual and interpretative discipline, exemplifies this interplay. Integrating such models highlights cognitive robustness not merely as innate talent, but as a trainable skill that can be actively fostered through education and practice.

## 3 Conclusion and Outlook

Dermatopathology requires both standardization and individual judgment. Cognitive robustness complements formal criteria by highlighting experience, intuition, and metacognitive skill—especially in complex, ambiguous cases. Though not measurable, it offers a valuable framework for improving diagnostic reasoning under uncertainty.

## Ethics Statement

The author has nothing to report.

## Conflicts of Interest

The author declares no conflicts of interest.

## Data Availability

Data sharing not applicable to this article as no datasets were generated or analyzed during the current study.
